# Absence of the neutrophil serine protease cathepsin G decreases neutrophil granulocyte infiltration but does not change the severity of acute pancreatitis

**DOI:** 10.1038/s41598-019-53293-0

**Published:** 2019-11-14

**Authors:** Ali A. Aghdassi, Daniel S. John, Matthias Sendler, Christian Storck, Cindy van den Brandt, Burkhard Krüger, Frank Ulrich Weiss, Julia Mayerle, Markus M. Lerch

**Affiliations:** 1 Department of Medicine A, University Medicine Greifswald, 17475 Greifswald, Germany; 2 Division of Medical Biology, University of Rostock, 18051 Rostock, Germany; 3 Department of Medicine II, Ludwigs-Maximilians University Munich, 80539 Munich, Germany

**Keywords:** Acute pancreatitis, Health sciences

## Abstract

Acute pancreatitis is characterized by an early intracellular protease activation and invasion of leukocytes into the pancreas. Cathepsins constitute a large group of lysosomal enzymes, that have been shown to modulate trypsinogen activation and neutrophil infiltration. Cathepsin G (CTSG) is a neutrophil serine protease of the chymotrypsin C family known to degrade extracellular matrix components and to have regulatory functions in inflammatory disorders. The aim of this study was to investigate the role of CTSG in pancreatitis. Isolated acinar cells were exposed to recombinant CTSG and supramaximal cholezystokinin stimulation. In CTSG^−/−^ mice and corresponding controls acute experimental pancreatitis was induced by serial caerulein injections. Severity was assessed by histology, serum enzyme levels and zymogen activation. Neutrophil infiltration was quantified by chloro-acetate ersterase staining and myeloperoxidase measurement. CTSG was expessed in inflammatory cells but not in pancreatic acinar cells. CTSG had no effect on intra-acinar-cell trypsinogen activation. In CTSG^−/−^ mice a transient decrease of neutrophil infiltration into the pancreas and lungs was found during acute pancreatitis while the disease severity remained largely unchanged. CTSG is involved in pancreatic neutrophil infiltration during pancreatitis, albeit to a lesser degree than the related neutrophil (PMN) elastase. Its absence therefore leaves pancreatitis severity essentially unaffected.

## Introduction

Acute pancreatitis is one of the most common non-neoplastic gastrointestinal disorders and its incidence is rising^1,2^. Early intracellular digestive protease activation is considered to initiate acute pancreatitis followed by an infiltration of inflammatory cells into the pancreas which exacerbates the disease. Trypsin is one of the earliest digestive enzymes undergoing activation by limited proteolysis and it further activates other proteases in a cascade like manner^3,4^. Lysosomal enzymes, specifically cathepsin B (CTSB), D (CTSD), and L (CTSL) either directly or indirectly regulate trypsinogen activation. While CTSB directly cleaves trypsinogen into its active form^5^, CTSD proteolytically activates pro-CTSB leading to a subsequent trypsinogen activation^6^. CTSL counteracts the effect of CTSB and degrades both trypsinogen and active trypsin^7^. Recently it could be shown that cathepsin C can also modulate the severity of pancreatitis, not by participating in intra-acinar cell zymogen activation but by interfering with the infiltration of neutrophil granulocytes into the pancreas. The underlying mechanism is the cleavage of the cell adhesion molecule E-cadherin by the serine protease neutrophil elastase (NE), which is activated by CTSC, thus facilitating inflammatory cell invasion^8^.

Cathepsin G (CTSG) is a serine protease of the chymotrypsin C family stored in its active form in azurophilic granules of neutrophil granulocytes together with NE and proteinase 3 (PR3). At the site of an inflammatory process neutrophil enzymes are released by neutrophil granulocytes. CTSG is activated by removal of a pro-peptide mediated by CTSC^9^. Biological functions of CTSG include degradation of extracellular matrix and plasma proteins. By cleavage of chemokines and their receptors CTSG also modulates immune functions^10^. CTSG is further involved in detachment of adhering leukocytes from the endothelium and their transmigration into damaged tissue^11^. It also localizes to neutrophil extracellular traps, a mechnism recently found to be of relevance in pancreatitis^12^.

In view of the different interactions of CTSG under inflammatory conditions we investigated whether this serine protease also exerts effects on acute pancreatitis using a knockout mouse model for CTSG. Although CTSG reduces invasion of neutophil granulocytes this effect was not sufficient to decrease the severity of acute pancreatitis, giving it an inferior role to neutrophil granulocyte enzymes such as PNM elastase. These results suggest only a subordinate role of CTSG in progression of acute pancreatitis.

## Results

### CTSG expression in leukocytes and pancreas

**Figure 1:**
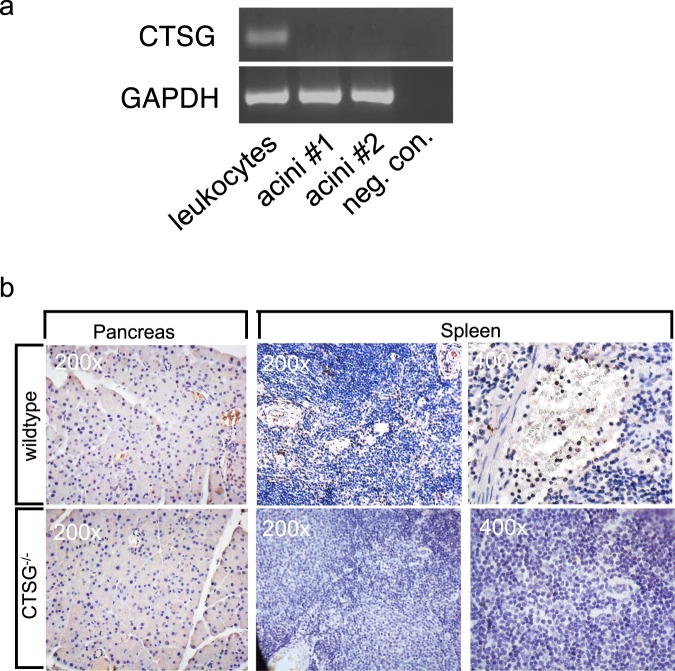
Cathepsin G is expressed in leukocytes but not in pancreatic acinar cells. (**a**) Leucocytes extracted from spleen express CTSG as shown by RT-PCR. No expression was detected in acinar cells investigated in two different samples (#1 and #2). GAPDH served as a loading control. In the negative control only water was used. (**b**) Cells with positive labelling for CTSG were found inside blood vessels that contain leukocytes as shown on 400-fold magnification in the spleen tissues. Pancreatic acinar cells were deficient of CTSG. Specificity of CTSG was proven in CTSG^−/−^ tissues that showed no labelling. Full length gels are presented in Supplementary Figs 2 and 3.

Because of the known CTSG expression in leukocytes we used this cell population as a positive control. In acinar cells no CTSG expression was detected by reverse transcriptase PCR. GAPDH was used as a loading control to show equal cDNA input (Fig. 1a). Our observations were confirmed by immunohistochemistry of pancreatic tissues of wildtype mice, in which no CTSG was detected in pancreatic acinar cells (Fig. 1b). In contrast, CTSG expressing cells were found in spleen tissues especially in cells within the vessels of wildtype mice, while no expression was found in CTSC^−/−^ spleens, as expected. Our findings indicate that CTSG expression is absent in excretory pancreatic cells.

### Effects of CTSG on trypsinogen processing *in-vitro* and protease activation, and cellular necrosis in acinar cells

**Figure 2:**
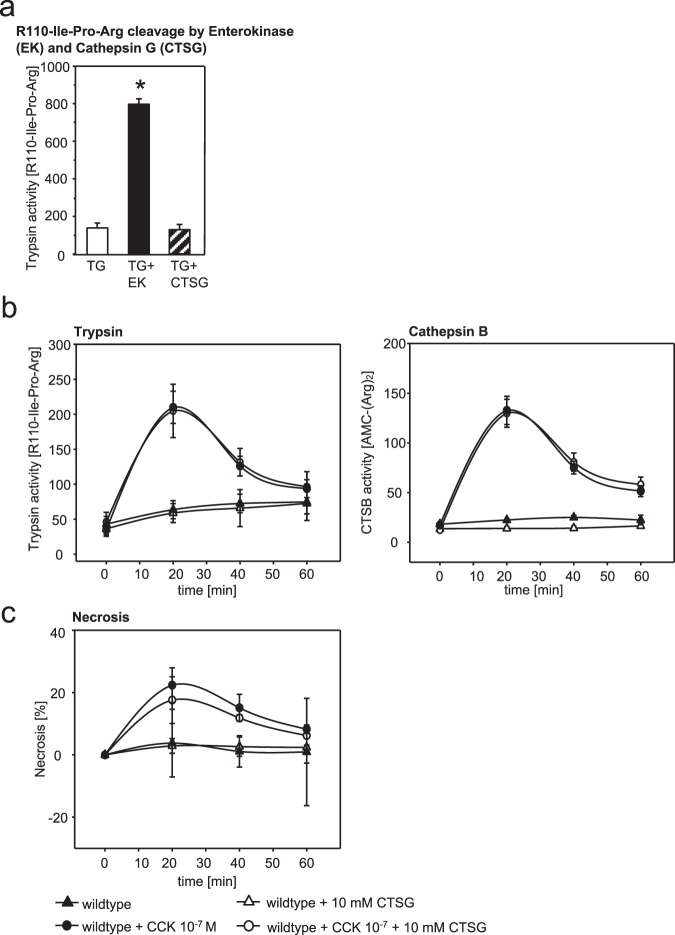
CTSG does not activate trypsinogen *in vitro* or in isolated acinar cells. (**a**) Addition of enterokinase *in vitro* rapidly increased trypsinogen (TG) activation as measured by R110-Ile-Pro-Arg cleavage, whereas CTSG had no such effect. (**b**) Intracellular trypsinogen and CTSB activation were not affected by addition CTSG to acinar cells that were supramaximally stimulated with CCK. (**c**) Cellular necrosis, measured by propidium iodide inclusion did not change after addition of CTSG. Shown are means +/− SEM. *Denotes p < 0.05. Pooled data from n = 5 mice.

To examine whether CTSG has a direct effect on the cleavage of trypsinogen *in vitro* we co-incubated trypsinogen with active CTSG *in vitro* and measured active trypsin activity by using the substrate R110-(CBZ-Ile-Pro-Arg)_2_. Even high CTSG concentrations did not lead to an increase in trypsin activity in contrast to enterokinase, a known trypsinogen activator, that induced a rapid increase in trypsin activity (Fig. 2a). In order to clarify whether CTSG has a direct effect on intracellular trypsin activation *in vivo* we isolated pancreatic acinar cells and co-incubated with recombinant CTSG and supramaximal doses of cholecystokinin. One fraction was treated with CTSG alone. In preliminary experiments we had shown that enzymatic CTSG activity is still present in the cultivation media of acinar cells (data not shown). Supramaximally CCK-stimulated acinar cells showed an increase of intracellular trypsin activity peaking at 20 minutes while the addition of CTSG, even at a high concentration (10 mM), did not affect trypsinogen activation. Intracellular cathepsin B (CTSB), a known trypsinogen activator, also rapidly increased in its activity within the first 20 minutes after CCK-stimulation and steadily decreased thereafter. It did not show any change of activity in the presence of CTSG (Fig. 2b). Moreover, the extent of cellular necrosis, measured by propidium iodide (PI) exclusion, was not altered by addition of CTSG (Fig. 2c). These results indicate that early protease activation in acinar cells and cell injury are independent of CTSG.

### Decreased neutrophil invasion in CTSG^−/−^ mice during acute pancreatitis

**Figure 3:**
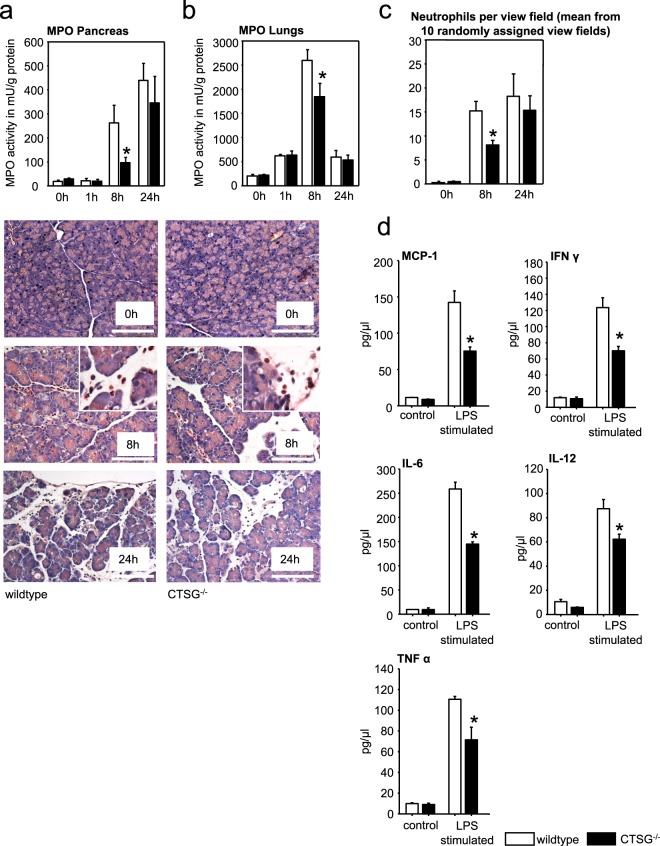
Neutrophil infiltration is transiently decreased in the absence of CTSG. (**a**) Decreased MPO activity in pancreas homogenates of CTSG^−/−^ mice at 8 h during caerulein-induced pancreatitis, which was no longer evident after 24 h. (**b**) MPO activity was decreased in CTSG^−/−^ lung tissue homogenates at 8 hours and returned to almost normal values at 24 hours. (**c**) Chloro-acetate-esterase staining in pancreas tissue indicated a transiently decreased infiltration of neutrophil granulocytes into the pancreas of CTSG^−/−^ mice at 8 hours when compared to wildtypes, which was similar again at 24 hours. (**d**) Reduced secretion of cytokines MCP-1, IFN γ, IL-6, IL-12, and TNF-α in isolated and LPS stimulated CTSG^−/−^ neutrophils. Shown are means +/− SEM. *Denotes p < 0.05. Scale bar, 50 μm. Pooled data from at least n = 4 mice.

Since CTSG is expressed in neutrophil granulocytes and is responsible for activation of several other neutrophil proteases^13^ we investigated whether depletion of CTSG would change the infiltration of neutrophils in the pancreas. Activities of myeloperoxidase (MPO), a neutrophil marker, increased during acute pancreatitis in pancreas homogenates showing the highest activities at 24 hours. In CTSG^−/−^ mice this increase was delayed leading to a significantly lower MPO activity at 8 hours, which was no longer evident at 24 h (Fig. 3a). In the lungs MPO activities were highest at 8 hours and strongly decreased at 24 hours. In CTSG^−/−^ mice MPO activity was significantly lower at 8 hours (Fig. 3b). The observation of reduced MPO levels in the pancreas at 8 hours was confirmed by chloroacetate esterase staining of pancreatic tissue that revealed a lower number of stained neutrophil granulocytes in the pancreas of CTSG deficient mice at 8 hours (Fig. 3c). No significant differences were observed at 24 hours between wild type and knockout animals. Quantitative measurements of the pro-inflammatory cytokines MCP-1, IFN γ, IL-6, IL-12, and TNF-α in the supernatants of isolated and lipopolysaccharide (LPS) stimulated CTSG^−/−^ neutrophils showed reduced activities compared to wildtype cells (Fig. 3d). These results indicate that cytokine production in neutrophils is impaired in the absence of CTSG.

### Severity of acute pancreatitis in CTSG^−/−^ mice

**Figure 4:**
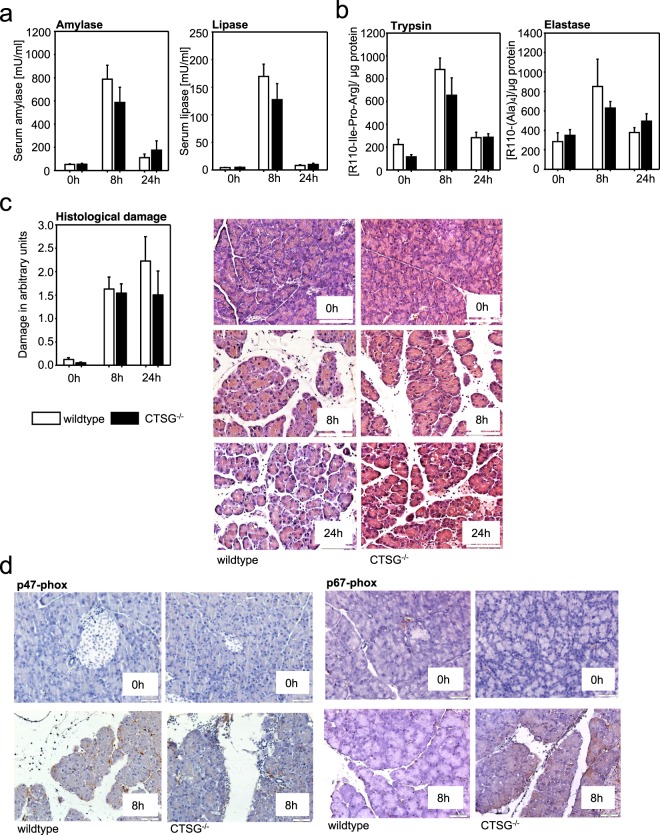
Absence of CTSG does not alter the severity of acute pancreatitis. (**a**) Serum amylase and lipase levels were strongly elevated at 8 hours of caerulein-induced pancreatitis but did not differ between 129S2/SvPasCrl (wildtype) and CTSG^−/−^ mice. (**b**) Trypsin and elastase activity were elevated at 8 hours but were independent of CTSG. **(c)** Histological damage was not altered in the absence of CTSG. (**d**) Staining of NCF-1 (p47-phox) and NCF-2 (p67-phox) in pancreatic tissue showed a clear positive signal for both proteins in wildtype as well as CTSG deficient mice after induction of pancreatitis. Shown are means +/− SEM. *Denotes p < 0.05. Scale bar, 50 μm. Pooled data from at least n = 4 mice.

To investigate whether depletion of CTSG alters the disease severity in acute pancreatitis mice we induced acute experimental pancreatitis using the caerulein-model. Both 129S2/SvPasCrl wild type and CTSG^−/−^ mice received serial intraperitoneal injections of the CCK-analogue caerulein. Serum lipase and amylase rapidly increased at 8 h, but did not differ significantly between wild type and CTSG knock out animals (Fig. 4a). At 8 hours the digestive proteases trypsin and elastase were increased in pancreatic tissue but not significantly different in CTSG deficient mice (Fig. 4b). Local pancreatic injury, determined by histological damage, consisting of vacuolization, necrosis, and inflammatory cell infiltration, increased during acute pancreatitis but was independent of the presence of CTSG, as no significant changes were observed between the groups (Fig. 4c). It is well known that neutrophil granulocytes increase pancreatic damage by release of reactive oxygen species (ROS)^14^. Neutrophil cytosolic factor 1 (NCF-1) and NCF-2 are part of the NADPH oxidase pathway and are responsible for the production of superoxide anions. A defect of NADPH oxidase mediated ROS production in infiltrating neutrophils could not be observed in our model. Wildtype mice as well as CTSG deficient animals show a clear positive signal for NCF-1 (p47-phox) or NCF-2 (p67-phox) in infiltrating neutrophils (Fig. 4d). These results indicate that the course and severity of pancreatitis do not depend on CTSG expression in mice, despite the transient decrease of neutrophil invasion into the pancreas seen at 8 hours (Fig. 3). Moreover, serum activities of cytokines were not significantly altered at 8 hours in CTSG knockout mice. Apparently systemic cytokine levels depend on more sources than only neutrophil granulocytes (Supplementary Fig. 1).

## Discussion

Lysosomal enzymes are important regulators in the early phase of acute pancreatitis. They can intracellularly activate as well as degrade the zymogen trypsinogen which is physiologically activated by the brush border enzyme enterokinase in the duodenum^15^. Once proteolysed to its active form trypsin causes an activation of multiple other digestive proteases which leads to cellular injury, ultimately resulting in pancreatitis^16^. During the initial events digestive enzymes and lysosomal hydrolases co-localize in the same subcellular compartment of acinar cells with subsequent release of active trypsin to the cytosol^4^. In addition to acini, trypsinogen activation also occurs in macrophages that harbor CTSB and ingest acinar cell components including trypsinogen once they have infiltrated the pancreas^17^.

Subsequently, inflammatory cells are recruited to the pancreas that further aggravate the severity of pancreatitis. The two main cell populations entering the pancreas are neutrophil granulocytes and macrophages^14,17^. This infiltration already takes place at early time points, even before pancreatic necroses have evolved^18^. Tumor necrosis factor α (TNF-α) is one of the key mediators that cause trypsin activation and pancreatic injury. TNF-α is released by immune cells, and its inhibition^3^ or deletion of the corresponding receptor^19^ abrogates disease severity. The TNF-α mediated trypsin activation is dependent on CTSB^3^. Furthermore, nicotinamide adenine dinucleotide phosphate (NADPH) oxidase regulates trypsin activation by generating reactive oxygen species making trypsinogen more susceptible to hydrolysis by serine proteases^14^.

Recently CTSC was identified as a lysosomal hydrolase, whose deficiency ameliorated the severity of pancreatitis^8^. CTSC processes the neutrophil serine proteases elastase (NE), proteinase 3 (PR3) and cathepsin G (CTSG), which are stored in primary neutrophil granules and participate in the progression of many inflammatory disorders via a variety of mechanisms^20^: One of their primary functions is to degrade engulfed microorganisms but they are also released directly at sites of inflammation and play an important role in cell signalling, degradation of extracellular matrix and processing of cytokines^21,22^.

The role of neutrophil elastase (NE), syn. polymorphonuclear (PMN) granulocyte elastase, in acute pancreatitis has been investigated in earlier studies that showed its ability to cleave the cell contact molecule E-cadherin and to facilitate the dissociation of cell contacts, which than permits an increased transmigration of leukocytes into the pancreas. Conversely, elastase inhibition significantly reduced the translocation of leukocytes and decreased the disease severity^23^.

The impact of other serine proteases in acute pancreatitis is incompletely understood. CTSG is abundantly expressed in neutrophils which makes this enzyme an interesting candidate. Our study showed, that its absence had no effect on the severity of pancreatitis despite a reduction of neutrophil invasion into the pancreas. The latter was transient. Moreover, protease activation in acinar cells, a hallmark of early pancreatitis, was independent of CTSG.

One explanation for the reduced neutrophil invasion into the pancreas of CTSG^−/−^ mice might be the capacity of CTSG to cleave vascular endothelial (VE)-cadherin, a cell contact protein expressed on the surface of the endothelial cells in blood vessels^24^. Absence of CTSG will maintain the barrier of the endothelial cells and thus impair the invasion of neutrophil granulocytes. In contrast, the adherens junction protein E-cadherin between acinar cells is not substantially cleaved by CTSG as we could show previously and dissociation of acinar cells was independent of presence of CTSG^8^. These findings may explain why the severity of acute pancreatitis is not ameliorated in CTSG^−/−^ mice compared to the wildtype.

Another possible explanation would be, that the moderately reduced number of neutrophils is not sufficient to improve the course of acute pancreatitis. Furthermore, this reduction occurred only transiently at 8 hours, and was no longer evident at 24 hours. The inflammatory infiltrate also consists of invading macrophages, which ingest digestive zymogens from injured acinar cells and activate zymogens in a CTSB dependent manner^17^. As long as macrophages are entering the pancreas, perpetuation of acute pancreatitis occurs due to persistent trypsinogen activation. Despite a reduced secretion of pro-inflammatory cytokines in isolated CTSG^−/−^ neutrophils *in vitro* systemic cytokine levels in serum were not significantly different between wildtype and CTSG knockout mice. Other inflammatory cells appear to contribute to a greater extend to cytokine secretion which could make the reduced cytokine release from CTSG^−/−^ neutrophils clinically irrelevant. In addition to mast cells and macrophages, also lymphocytes, including B- and T-cells and NK-cells are known to have a role in systemic inflammation^25–27^.

Mice deficient in both neutrophil elastase and cathepsin G have been investigated in a previous study, that showed a normal recruitment of neutrophils to the site of inflammation upon induction of sterile peritonitis. In addition, normal TNF-α levels were produced in these mice^28^. These findings are in line with our observations as acute pancreatits is a primarily sterile infection. However, susceptibility to fungal infections was more severe in double NE and CTSG knockout mice^28^. CTSG^−/−^ mice were also investigated after infection with Mycobacterium bovis bacillus Calmette-Guérin (BCG) and showed an impaired pathogen elimination^29^.

Rats that underwent ischemia and reperfusion (I/R)-induced pancreatitis and were treated with Lex032, a broad serine protease inhibitor, preserved capillary perfusion and showed reduced serum IL-6 and amylase concentrations. These results indicate that serine protease inhibition in general has an anti-inflammatory potential^11^ but probably CTSG inhibtion alone is not sufficient to alter the severity of the disease. However, comparability of these results is challenging, as the authors used a different experimental model for acute pancreatitis than in our study.

In conclusion, our results indicate that the absence of cathepsin G leads to a transient reduction in neutrophil granulocyte invasion but leaves pancreatitis severity largely unaffected. This effect, however, is less prominent than for the related protease PNM elastase^23^, or the calcium binding protein S110A9^18^, both of which facilitates leucocyte infiltration via cleavage of cell-cell contacts between pancreatic acinar cells, which CTSG does not. The former therefore represent more promising treatment targets than CTSG.

## Materials and Methods

### Reagents, substrates and antibodies

Collagenase from *Clostridium histolyticum* (EC.3.4.24.3) was purchased from SERVA (lot no. 14007, Heidelberg, Germany) and used for acinar cell isolation. Cathepsin G enzyme (human neutrophil) was obtained from Merck (Darmstadt, Germany). Myeloperoxidase (MPO) enzyme from human polymorphonuclear leukocytes, enterokinase from porcine intestine, caerulein, and cholecystokinin (CCK) were obtained from Sigma (Taufkirchen, Germany). Ketamine and xylazine were from Selectavet (Weyarn-Holzolling, Germany). Amylase and lipase were quantified using a kit from Roche-Hitachi (Grenzach-Wyhlen, Germany). Anti-cathepsin G (ab64891) was purchased from abcam (Cambridge, UK). Anti-p47-phox (sc-17844) and anti-p67-phox (sc-374510) were purchased from Santa Cruz Biotechnology (Dallas, TX). Protease activity was measured by adding the following substrates: for trypsin R110-(CBZ-Ile-Pro-Arg)_2_ from Invitrogen (Carlsbad, CA), for cathepsin B (AMC-Arg_2_) from Bachem (Bubendorf, Switzerland), and for elastase (CBZ-Ala-Ala-Ala-Ala)_2_-R110 from Molecular Probes (Waltham, MA, USA). Substrate for caspase-3 (R110-DEVD) was obtained from Invitrogen (Carlsbad, CA, USA).

### Chloro-acetate esterase staining

Staining solution was prepared by adding 3 drops (150 μl) of 4% p-rosanilin in 2 M hydrochoric acid, 3 drops of sodiumnitrite (4%) in 90 ml PBS and addition of 30 mg naphthol-AS-D-chloroacetate with 10 drops (500 μl) of dimethylformamide. The solution was mixed well and filtered. Pancreatic tissue sections were deparaffinazed and were incubated with the chloro-acetate esterase staining solution for 60 min at 37 °C. After intensive rinsing slides were counterstained with hematoxilin, rinsed in water and mounted in Crystal Mount TM Aqueous Mounting Medium (Sigma-Aldrich, Taufkirchen, Germany) and finally in Pertex ® mounting medium (VWR International, Darmstadt, Germany). Scoring of infiltrating cells were performed by counting numbers of cells per visual field. A minimum of 10 visual fields for each animal were quantified

### Immunohistochemical staining of p47 and p67

2 µm thick slides of paraffin embedded tissue were used for immunohistochemical staining. Antigen retrieval was performed by cooking the slides in DAKO Antigen retrieval buffer (DAKO, Santa Clara, CA) for 30 min. Endogeneous peroxidase activity was blocked by 3% H_2_O_2_. Slides were blocked with PBS containing 5% bovine serum albumine (BSA) and 0.3% Triton X-100 for 1 h. Antibodies were diluted in PBS containing 1% BSA and 0.3% Triton X-100 and added on the slides and incubated over night at 4 °C. Anti-mouse secondary antibody was used from DAKO. DAB staining kit was used from Vector Laboratories (Burlingame, CA). After DAB reaction the slides were counterstained with Hematoxylin and mounted with VectaMount mounting media from Vector Laboratories.

### Reverse transcriptase PCR

Purification of RNA was done using TRIzol reagent (Thermo Fisher Scientific, Waltham, MA) from isolated acinar cells and leukocytes from spleen tissue. Single strand cDNA synthesis was generated from 500 ng RNA as starting material using the Reverse Transcriptase System from Promega (Mannheim, Germany). For cathepsin G (NM_007800) the following gene specific primers were used: sense primer: 5′-GAG TCC AGA AGG GCT GAG TG-3′ and antisense primer: 5′-CCT TTC TCG CAT TTG GAT GT-3′ amplifying a 136 bp fragment^30^. Glyceraldehyde-3-phosphate dehydrogenase (GAPDH) was used as a reference for equal cDNA input. The PCR mixture contained 2x FastStart Master Mix (Roche, Basel, Switzerland), primers (200 nM each), cDNA (approximately 100 ng) in a final volume of 50 μl. Amplification was carried out in an Eppendorf cycler for 35 cycles, annealing temperatures were 62 °C for CTSG primers and 60 °C for GAPDH primers. Negative controls without DNA were set up for each PCR reaction. PCR products were visualized under ultraviolet light on a 2.0% agarose gel containing ethidium bromide.

### Induction of acute pancreatitis in mice

All animal experiments were performed according to state institutional guidelines and animal facility protocols after prior approval by the institutional animal care committee. Wildtype 129S2/SvPasCrl wildtype mice were obtained from Charles River Laboratories (Sulzfeld, Germany). CTSG^−/−^ mice [129-CtsgG^tm1.1Roes/(H)^] were purchased from the European Mouse Mutant Archive (EMMA). Acute pancreatitis was induced by hourly intraperitoneal injections of caerulein (50 µg/kg/body weight) for up to 8 hours^31^. All mice were starved over night with access to water ad libitum. Mice were sacrified at 0 h, 8 h, and 24 h after the first caerulein injection.

Tissue was harvested immediately after sacrificing the animals. Pancreas and lung samples were frozen in liquid nitrogen and stored at −80 °C for later analysis. For histology, tissue was fixed in 4.5% formalin for paraffin embedding. Serum samples were and stored at −20 °C.

### Isolation of Acinar Cells

Acinar cells were prepared by collagenase digestion as previously described^32^. Cells were maintained and stimulated in Dulbecco’s modified Eagle’s medium containing 10 mM 4-(2-hydroxyethyl)-1-piperazine ethanesulfonic acid (HEPES) and 2% bovine serum albumin (BSA) with 1 µM cholecystokinin (CCK). One fraction of acinar cells was co-incubated with 10 mM CTSG enzyme. Protease activity was monitored for up to 60 min after CCK stimulation and aliquots were taken every 20 minutes. Untreated cells served as controls. *In vivo* measurement of protease activation in living acinar cells was performed in cell medium (pH 7.4) containing 24.5 mM HEPES, 96 mM NaCl, 11.5 mM glucose, 6 mM KCl, 1 mM MgCl_2_ 6H_2_O, 0.5 mM CaCl_2_ 2H_2_O, 2.5 mM NaH_2_PO_4_ H_2_O, 5 mM sodium fumarate, 5 mM sodium glutamate, 5 mM sodium pyruvate, and 1% BSA and DMEM in a flurometer (FLUOStar Omega, BMG Labtech GmbH, Ortenberg, Germany) as described previously^8^. For trypsin activity the substrate R110-(CBZ-IPR)_2_ (10 µM), and for CTSB activity the substrate AMC-Arg_2_ (20 µM) were used. Necrosis was determined by measurement of propidium iodide incorporation in acinar cells. All measurements were done in triplicates.

### Histology and immunohistochemistry

Paraffin sections from the pancreas and spleen were used for haematoxylin and eosin (H&E) staining. For assessment of damage a modified score, adapted from Niederau *et al*., was used^33^. The scoring was based on the extent of necrosis, vacuolization of acinar cells and invasion of inflammatory cells into the pancreas. A minimum of 10 visual fields from each animal was investigated. For immunohistochemistry antigen retrieval was performed with Target Retrieval Solution (Dako). CTSG was stained using an anti-cathepsin G antibody (ab64891), diluted 1:50 in 20% fetal calf serum (FCS) from PAN Biotech (Aidenbach, Germany) and incubated over night at + 4 °C followed by staining with a peroxidase-HRP labeled antibody (1:1000 dilution). CTSG stained cells were visualized using the DAB (3,3′-diaminobenzidine) Substrate Kit (Vector Laboratories, Burlingame, CA, USA) according to the manufacturer’s instructions.

### Isolation of neutrophils

Leukocytes were isolated from murine spleens of CTSG^−/−^ and WT mice as described previously^8^. Briefly, after passing the cell suspension over a 70 μm sterile nylon filter (BD Falcon, Amsterdam, The Netherlands) to remove cell aggregates and debris erythrocytes were lysed with 155 mM NH_4_Cl, 10 mM KHCO_3_, and 0.1 mM EDTA^34^. Neutrophils were isolated from total leukocytes using MACS^®^ cell separation kit according to the manufacturer’s instructions (Miltenyi Biotech, Bergisch-Gladbach, Germany) by using Ly6g as a specific antibody.

### Biochemical assays

Serum lipase and amylase activities were measured by photometric assays (Roche Hitachi, Grenzach-Whylen, Germany) as kinetics over 30 min with an absorbance at 570 nm at 37 °C. Purified enzymes (purchased from Sigma) served as controls. Trypsin and elastase activity were determined from pancreatic tissue homogenates and were measured at 37 °C in a buffer containing 100 mM Tris, 5 mM CaCl_2_ pH 8.0, by adding 10 µM of R110-Ile-Pro-Arg for Trypsin or (CBZ-Ala-Ala-Ala-Ala)_2_-R110 for elastase as enzyme kinetic over 1 h at 37 °C at an excitation wavelength of 485 nm and an emission wavelength of 520 nm.

For *in vitro* proteolytic cleavage of trypsinogen either enterokinase (40 mU/ml) or cathepsin G enzyme (50 mU/ml) were added to 0.05 pg/ml bovine trypsinogen and incubated for 1 hour. Active trypsin was measured as enzyme kinetic by adding 10 µM of R110-Ile-Pro-Arg in 100 mM Tris, 5 mM CaCl_2_ pH 8.0 buffer.

Myeloperoxidase (MPO) activity measurement was performed as previously described^3^. Briefly pancreatic tissue was homogenized on ice in 20 mM potassium phosphate buffer (pH 7.4) and centrifuged at 20,000 x g at 4 °C for 10 min. The pellet was resuspended in 50 mM potassium phosphate buffer (pH 6.0) containing 0.5% cetyltrimethylammonium bromide. The suspension was frozen in liquid nitrogen and thawed in cycles, sonicated twice and centrifuged at 20,000 x g at 4 °C for 10 min. MPO activity was measured in 50 mM potassium phosphate buffer (pH 6.0) containing 0.53 mM O-dianisidine and 0.15 mM H_2_O_2_. The initial increase in absorbance was measured at room temperature with a Spectramax Spectrophotometer (Molecular Devices, Sunnyvale, CA). The results were expressed in units of MPO activity on the basis of 1 unit being able to oxidize 1 μmol H_2_O_2_ per minute per milligram pancreatic protein^32^. Cytokines TNF-α, IL-6, IL-12, IFN γ, and MCP-1 were measured from serum samples by FACS analysis with the CBA mouse-inflammation-kit according to the manufacturer’s instructions (Becton Dickinson, Heidelberg, Germany).

### Statistical analysis

All data are expressed as means ± SEM from at least four animals per experiment in each group. Statistical analyses were performed by SigmaPlot (Systat Software Inc, Erkrath, Germany) and SigmaStat (Systat Software Inc, Erkrath, Germany) using the unpaired 2-tailored student t-test or analysis of variances (ANOVA) for samples without normality. Differences were considered significant for p < 0.05.

### Ethical approval and informed consent

All experimental protocols were approved by the institutional animal care committee (Landesamt für Landwirtschaft, Lebensmittelsicherheit und Fischerei Mecklenburg-Vorpommern).

## Supplementary information

Supplementary figure

## Data Availability

The datasets generated during and analyzed during the current study are available from the corresponding author on request.
