# Supplementary material for: Absence of the neutrophil serine protease cathepsin G decreases neutrophil granulocyte infiltration but does not change the severity of acute pancreatitis
Source: Sci Rep. 2019 Nov 14;9:16774. doi: 10.1038/s41598-019-53293-0 (PMC6856518; doi:10.1038/s41598-019-53293-0)
Supplement: Supplementary file 1 — Supplementary figure [file 41598_2019_53293_MOESM1_ESM.pdf]

Absence of the neutrophil serine protease cathepsin G decreases neutrophil granulocyte infiltration but does not change the severity of acute pancreatitis

Ali A. Aghdassi, Daniel S. John, Matthias Sendler, Christian Storck, Cindy van den Brandt, Burkhard Krüger, Frank Ulrich Weiss, Julia Mayerle, Markus M. Lerch

Supplementary figure 1

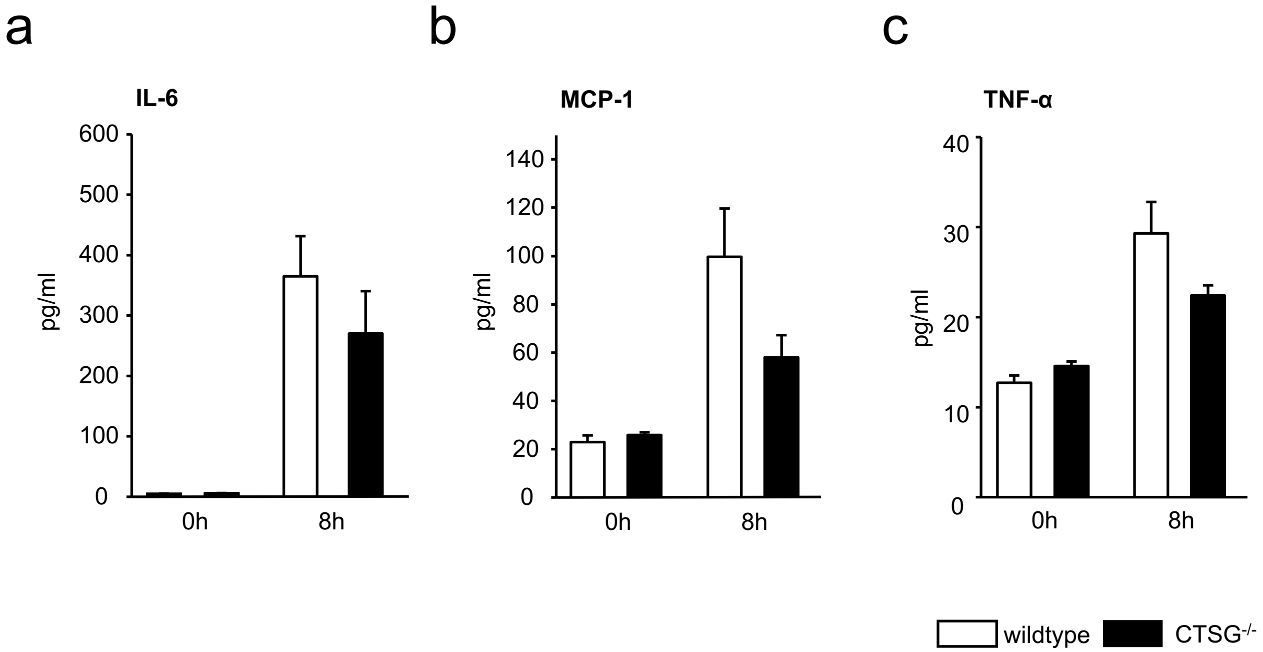

Supplementary figure 1. Serum cytokine concentrations are not significantly altered in CTSG$^{-/-}$ mice. There was a trend towards a reduced serum concentration of (a) IL-6, (b) MCP-1, and (c) TNF-α at 8 hours caerulein pancreatitis. Pooled data from at least n = 4 mice.

Supplementary figure 2

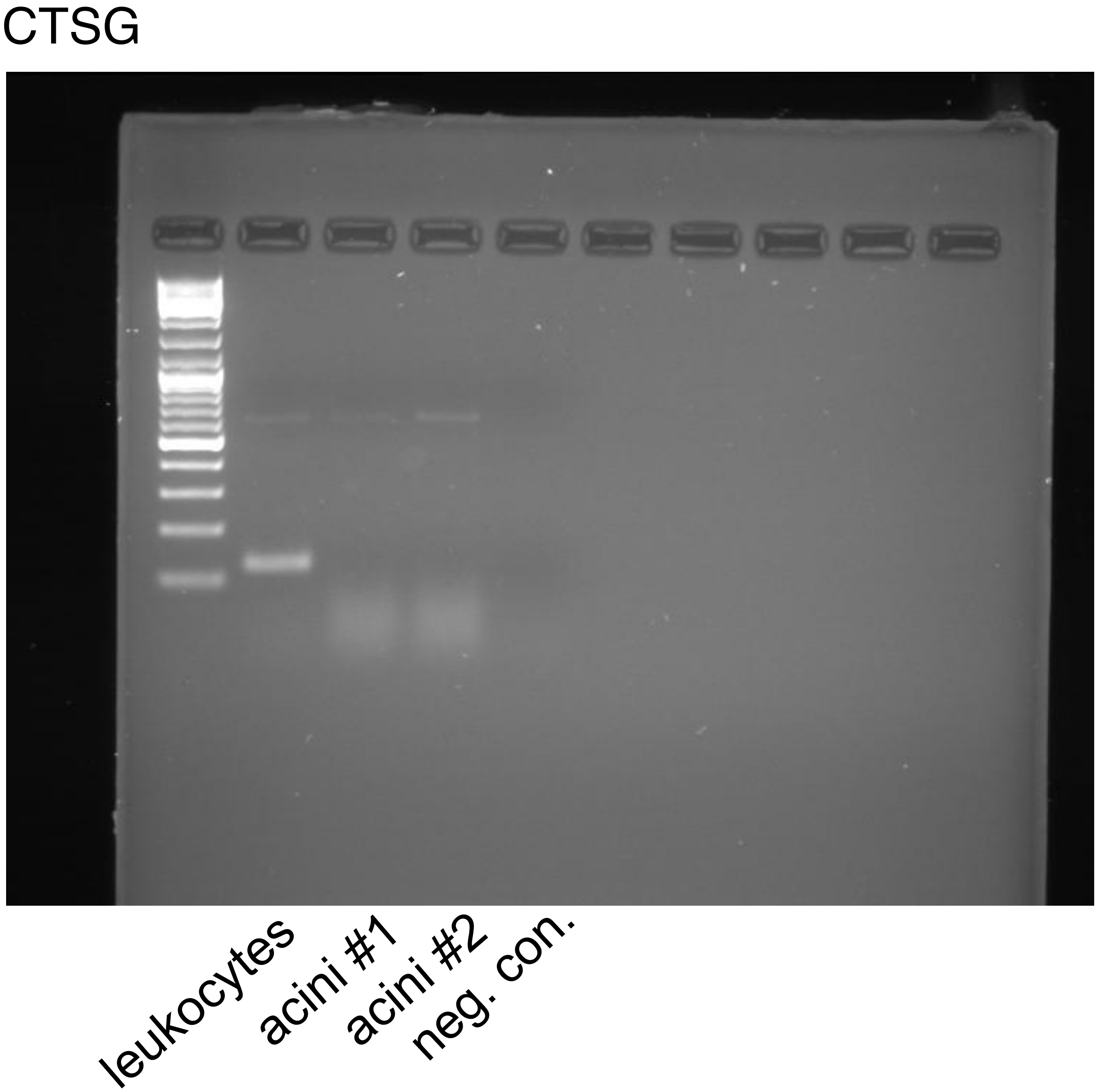

Supplementary figure 2. Full-length agarose gel of CTSG

Supplementary figure 3

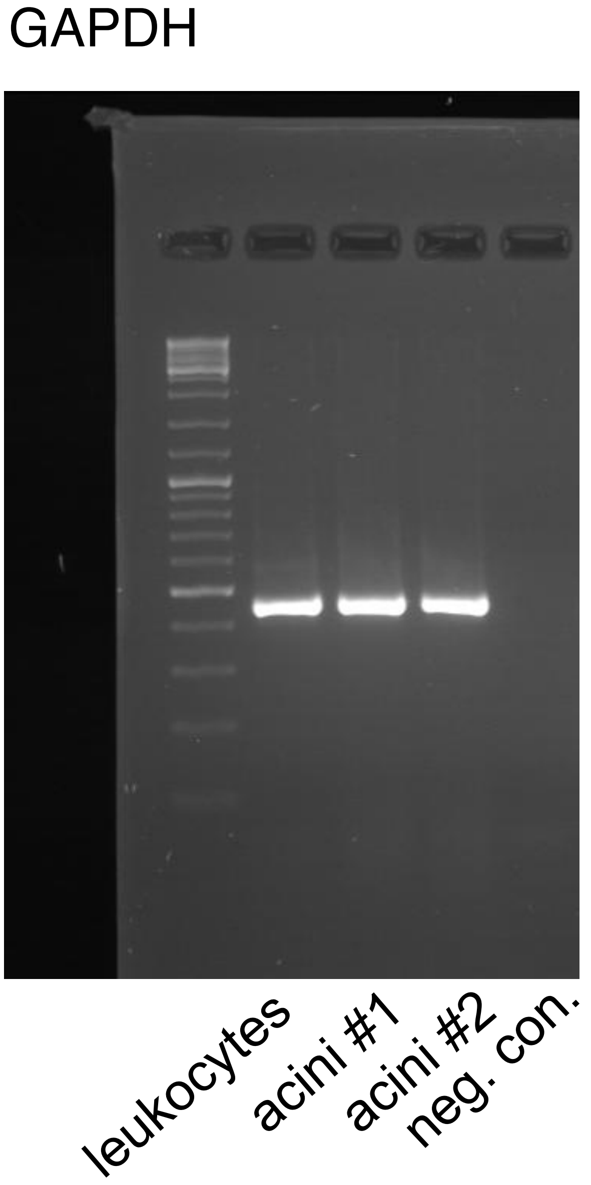

Supplementary figure 3. Full-length agarose gel of GAPDH
